# Commentary: SARS-CoV-2 Transmission in Patients With Cancer at a Tertiary Care Hospital in Wuhan, China

**DOI:** 10.3389/fonc.2020.01223

**Published:** 2020-07-10

**Authors:** Serena Di Cosimo, Luca Porcu, Andrea Malfettone, Javier Cortés, Rosalba Miceli

**Affiliations:** ^1^Biomarker Unit, Department of Applied Research and Technological Development, Fondazione IRCCS Istituto Nazionale dei Tumori di Milano, Milan, Italy; ^2^Laboratory of Methodology for Clinical Research, Department of Oncology, Istituto di Ricerche Farmacologiche Mario Negri IRCCS, Milan, Italy; ^3^Medica Scientia Innovation Research (MedSIR), New Jersey, United States; ^4^Medica Scientia Innovation Research (MedSIR), Barcelona, Spain; ^5^IOB Institute of Oncology, Quiron Group, Madrid, Spain; ^6^IOB Institute of Oncology, Quiron Group, Barcelona, Spain; ^7^Vall d'Hebron Insititute of Oncology (VHIO), Barcelona, Spain; ^8^Clinical Epidemiology and Trial Organization Unit, Department of Applied Research and Technological Development, Fondazione IRCCS Istituto Nazionale dei Tumori di Milano, Milan, Italy

**Keywords:** COVID-19, SARS-CoV-2 infection, cancer care, patient guidance, risk factors

## Introduction

Coronavirus disease 2019 (COVID-19), caused by the novel severe acute respiratory syndrome coronavirus 2 (SARS-CoV-2), has rapidly escalated to a global pandemic, triggering countries to implement lockdowns and stringent border controls. Under increasing pressures, healthcare institutions worldwide have taken precautions to attenuate the negative effects of the COVID-19 pandemic and continue to deliver optimal care to patients with cancer.

Thus, far, systematic reports about the incidence of asymptomatic COVID-19 in patients with cancer are very scarce. The most comprehensive data available at this time originates from a joint mission report indicating that in China the case fatality rate for patients with cancer was 7.6% compared with 1.4% for those with no comorbidities (1). Despite limited data, people with hematological and other cancers and who are undergoing active treatment with chemotherapy or radiotherapy seem to be at greater risk than others because their immune systems are compromised, they are of greater age, or they have multiple comorbidities. Moreover, patients with cancer often make frequent visits to the hospital for treatment and monitoring, presenting them with a heightened risk for exposure to SARS-CoV-2, possibly resulting in severe illness with COVID-19.

## Summary of the Report

We read with great interest the report in *JAMA Oncology* by Jing Yu and colleagues (2). Moving from recently reported results of a large epidemiologic study (3), the authors focused on the effect of the COVID-19 pandemic on hospitalized cancer patients at a single institution in Wuhan, China. A retrospective analysis of 1,524 patients with cancer revealed a higher incidence of SARS-CoV-2 infection compared with the community at large, though <50% were undergoing active anticancer treatments. Potential risk factors for COVID-19 transmission to patients attending hospitals for cancer care were also considered. In elderly patients (>60 years) with non-small cell lung cancer (NSCLC), the risk of contracting COVID-19 was 2.4 times higher than for younger patients (4.3 vs. 1.8%) even though no association with susceptibility to infection was found in a prior multicenter study of 1,099 patients with laboratory-confirmed COVID-19 (4).

The authors deserve credit and their effort is worth mentioning because establishing the extent of COVID-19 in a very vulnerable population could help guide formulation of policies to optimally reduce the spread of disease amid a pandemic. However, some of the methodology is not solid, giving rise to a lack of confidence in the results.

## Discussion

First, Jing Yu, and colleagues concluded that patients with cancer were at greater risk for SARS-CoV-2 infection compared with the Wuhan community at large. However, it appears that tests for systematic errors (i.e., selection bias [symptomatic people visit the hospital more often than uninfected/paucisymptomatic people], assessment bias [inconsistency between the two populations in the application of COVID-19 diagnostic methods], and sampling methods) were not conducted. In addition, the method for obtaining the odds ratio (OR) was not disclosed. By arranging the data in a 2x2 table, the OR is 2.13 (95% confidence interval [CI], 1.21–3.76).The authors reported that the OR was 2.31 (95% CI, 1.89–3.02). Perhaps this was calculated using a multivariable analysis with an adjustment for heterogeneity in demographics and COVID-19 risk factors between the two populations. By omitting an adequate description, the data do not provide repeatable results, presenting difficulties in interpretation.

Second, while Jing Yu et al. stated that cancer treatment is reportedly associated with immunosuppression, data showing a relationship between the use of chemotherapy, immunotherapy, or radiotherapy and SARS-CoV-2 infection are not provided. While it is true that almost half the patients with both COVID-19 and cancer were actively treated for the cancer, the distribution of various treatments among those with COVID-19 is not known to be comparable to that among those without it.

Last, Table 2 of the report by Jing Yu and colleagues (2) shows the association between age and comorbid COVID-19 in patients with NSCLC. It is likely that 60 years of age was chosen as the cutoff because it was convenient. This choice raises certain concerns, however. For example, the usual point at which elder age begins is 65 years. Also, advanced age is largely recognized as a risk factor for symptomatic COVID-19 regardless of cancer status (5). Finally, the reported association between age and the presence of COVID-19 was not significant (*p* = 0.447 using the Fisher exact test [unreported]) and, even so, association does not imply causation. Because this stated study limitation, that “older patients and patients with NSCLC may be at risk of COVID-19,” Table 2 data must be interpreted with caution.

## Conclusion

Overall, conclusive associations between contracting COVID-19 and receiving cancer care cannot yet be determined. Additional detailed and unbiased data describing how patients with cancer differ in susceptibility to COVID-19 and complications related directly to SARS-CoV-2 infection represent an unmet need of the oncologic community. Further studies are required to elucidate the clinical characteristics of SARS-CoV-2-infected patients before making statements or recommendations that might preclude or delay treatments of life-threatening diseases.

## Author Contributions

SDC, AM, and RM did the literature search and drafted the manuscript. SDC, LP, AM, JC, and RM put forward the hypothesis, revised the paper, and approved it for publication.

## Conflict of Interest

SDC is a member of a speakers bureau for Novartis and is an advisory board member for Pierre Fabre. JC has stock or other ownership in Medica Scientia Innovation Research (MedSIR); is a consultant/advisory board member for Roche, Celgene, Cellestia, AstraZeneca, Biothera Pharmaceutical, Merus, Seattle Genetics, Daiichi Sankyo, Erytech, Athenex, Polyphor, Lilly, Servier, Merck Sharp & Dohme, GSK, Leuko, Bioasis, and Clovis Oncology; has received honoraria from Roche, Novartis, Celgene, Eisai, Pfizer, Samsung Bioepis, Lilly, Merck Sharp & Dohme, and Daiichi Sankyo. Research funding was provided to Vall d'Hebron Insititute of Oncology (VHIO) from Roche, Ariad pharmaceuticals, AstraZeneca, Baxalta GMBH/Servier Affaires, Bayer healthcare, Eisai, F. Hoffman-La Roche, Guardant Health, Merck Sharp & Dohme, Pfizer, Piqur Therapeutics, Puma C and the Queen Mary University of London. AM was employed by the company Medica Scientia Innovation Research (MedSIR). The remaining authors declare that the research was conducted in the absence of any commercial or financial relationships that could be construed as a potential conflict of interest.
